# The association between different types of physical activity and smoking behavior

**DOI:** 10.1186/s12888-023-05416-1

**Published:** 2023-12-11

**Authors:** Jipeng Zhang, Yiwen Cao, Hongfei Mo, Rui Feng

**Affiliations:** 1 School of Physical Education (Main Campus), Zhengzhou University, Zhengzhou, Henan P.R. China; 2 College of Public Health, Zhengzhou University, Zhengzhou, Henan P. R. China

**Keywords:** Commuting physical activity, Recreation physical activity, Sedentary behavior, Smoking behavior, Work physical activity

## Abstract

**Background:**

Smoking is harmful, which has become a major public health burden. Physical activity may be related to smoking. Physical activity is one of the current methods for smoking control and smoking cessation. Different types of physical activity may have different effect on smoking behavior.

**Objective:**

The purpose of this study was to identify the direction and extent of the impact of different types of physical activity above moderate intensity (including work physical activity, recreational physical activity, commuter physical activity and sedentary behavior) on smoking behavior.

**Materials and methods:**

In this study, a total of 2,015 individuals (1,233 males and 782 females, mean age 54.02±17.31 years) was selected from the representative population aged 20 and above in the National Health and Nutrition Survey of the United States from 2017 to 2018. Physical activity was assessed using the Global Physical Activity Questionnaire (GPAQ) ; the tobacco use questionnaire (SMQ) was used to determine whether the sample had smoking behavior at this stage. Binary Logistic regression analysis was performed with various physical activities as independent variables and smoking behavior as dependent variables. All data were analyzed through Statistical Product and Service Solutions (SPSS) 26.0.

**Results:**

After adjusted for all confounding variables, physical activity at work was close to significantly associated with smoking behavior (*P*=0.053), odds ratio (OR) =1.135 (95%Cl: 0.999-1.289). Recreational physical activity was significantly associated with smoking behavior (*P* < 0.001), OR=0.729 (95%Cl: 0.639-0.832). Commuting physical activity was significantly associated with smoking behavior (*P* < 0.001), OR=1.214 (95%Cl:1.048-1.405). Sedentary behavior was significantly associated with smoking behavior (*P* < 0.001), OR=1.363 (95%Cl: 1.154-1.611).

**Conclusions:**

Given that different types of physical activity have different associations with smoking behavior. Therefore, when physical activity is used as a tobacco control measurement, it is necessary to pay attention to the type and environment of physical activity. Recreational physical activities should be appropriately increased, sedentary behavior should be reduced, and smoking prohibit environment should be expanded as far as possible to achieve better clinical intervention effects.

## Background

Smoking not only seriously harms physical and mental health, but also harms passive smokers, which has become one of the major public health problems. Even e-cigarettes have no small impact on health [1]. Smoking is one of the leading causes of preventable premature death worldwide, and according to a 2017 report by the World Health Organization (WHO), smoking kills more than 70,000 people each year [2]. However, about 21% of the world's population still smokes [3]. Commercially available cigarettes contain more than 7,000 chemicals, and their combustion creates potentially toxic substances in mainstream smoke, side-stream smoke, secondhand smoke, third-hand smoke, and discarded cigarette butts [4]. Smoking is associated with a number of fatal diseases and causes diseases of almost all organs of the body, including cancer, respiratory, cardiovascular, infectious and neurological diseases, leading to a decline in human health [5, 6]. Maternal smoking during pregnancy also affects fetal health and lung function [7]. In addition, both maternal smoking during pregnancy and paternal smoking were associated with overweight and obesity in adult daughters [8].

Adequate and appropriate physical activity enhances muscle and bone health, improves cardiovascular health, enhances immune system function, reduces anxiety, depression, and stress, and improves mood and well-being. Physical inactivity is a modifiable risk factor for cardiovascular disease and a variety of other chronic diseases, including diabetes, cancer, obesity, high blood pressure, bone and joint diseases, and anxiety and depression [9]. Anxiety and depression may lead to more smoking. At the same time, relevant studies have proved that physical activity is related to smoking behavior, and smokers tend to have less physical activity than non-smokers [10, 11], and exercise can quickly reduce the desire for cigarettes [12]. This may be related to the positive emotions that physical activity can produce, so physical activity is also currently used as a means of tobacco control. Given the dangers of smoking, the importance of physical activity to health, and the modifiable nature of physical activity and smoking behavior [13], it is important to explore the relationship between the two for effective tobacco control.

Although previous studies have confirmed the correlation between physical activity and smoking behavior, physical activity is not always beneficial and sometimes has negative effects, and inappropriate physical activity may cause damage to the body [14]. Kye [15] showed that high intensity physical activity at work was negatively correlated with obesity. Physical activity at work can have negative effects, while recreational physical activity in leisure time often leads to good emotional experiences [16]. Furthermore, physical activity is influenced by socio-demographic, biological, cognitive, emotional, sociocultural, and environmental factors [17]. Therefore, the intervention of physical activity on smoking behavior may not always be positive, and we suspect that different types of physical activity may have different associations with the effects of smoking behavior. Therefore, according to the different purpose of physical activity, this study divides it into four different types: work physical activity, recreational physical activity, commuting physical activity and sedentary behavior. The aim is to identify the direction and degree of correlation between different types of physical activity and smoking behavior, and put forward scientific targeted suggestions in daily physical activity, so as to control smoking more effectively to a certain extent.

## Methods and materials

### Object

**Fig. 1:**
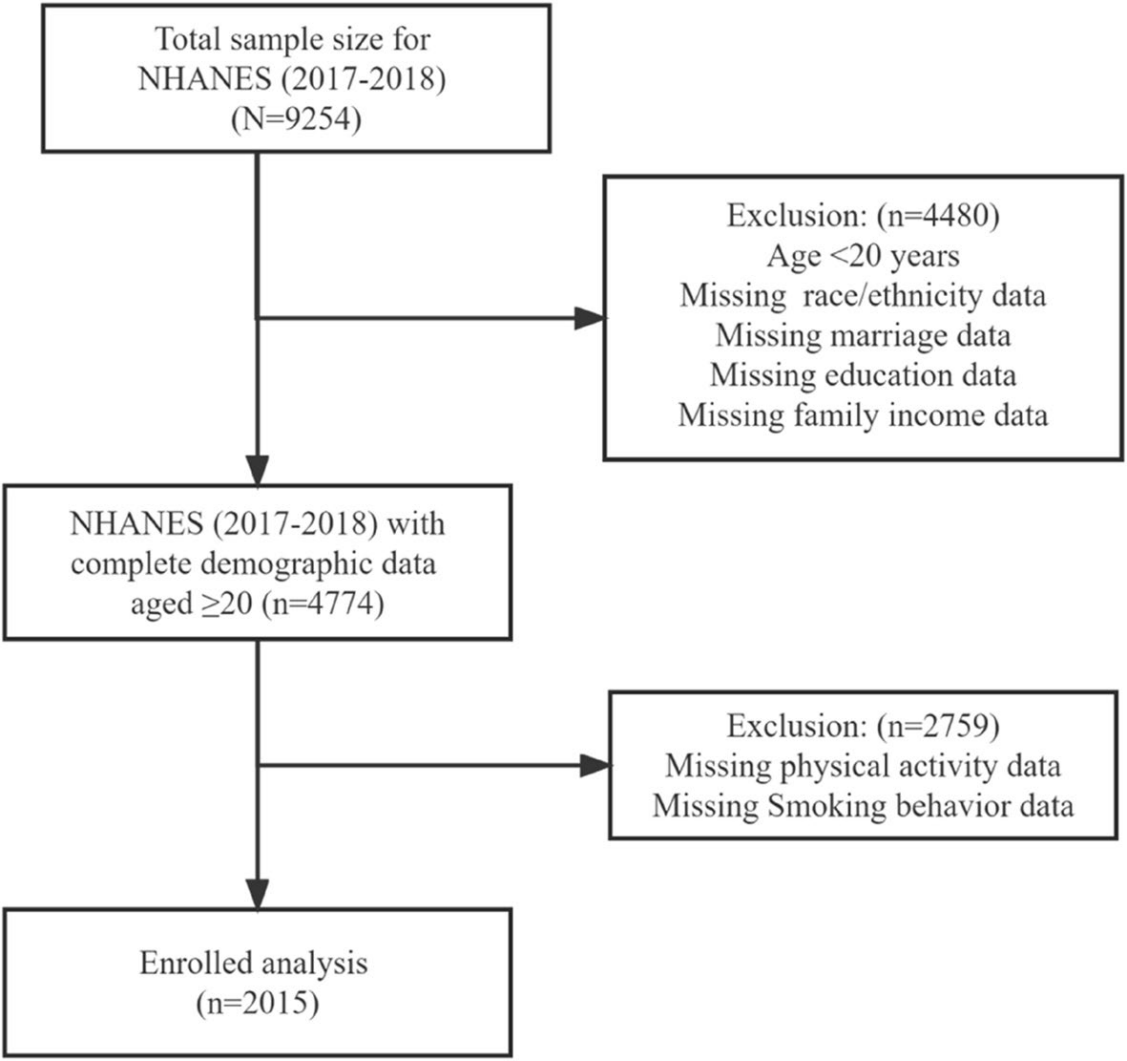
Data screening flow chart

The National Nutrition Examination Survey (NHANES) is a population-based, cross-sectional survey designed to collect information on the health and nutritional status of the U.S. household population [18]. This study used a representative sample of 2,015 individuals aged 20 years and older stratified by NHANES in 2017-2018. NHANES covers about 15,000 households, all of which are U.S. residents who have lived in the United States for at least two months. The survey protocol and secondary analyses of the data were approved by the Ethics Review Committee of the National Center for Health Statistics. All adult participants in NHANES gave informed consent to the purpose, risks and benefits of the study and signed an informed consent form [19]. Additional details on study design, sampling and exclusion criteria are shown in the figure below (See Fig. 1).

### Physical activity assessment

All types of physical activity (work physical activity, recreational physical activity, commuting physical activity, and sedentary behavior) were assessed using the GPAQ. Whether they engage in more than moderate intensity work physical activity, recreational physical activity, and commuting physical activity during the week. Sedentary duration <600min or ≥600min in a 24-hour period. The codes "1" and "2" indicate whether this type of physical activity or sedentary duration <600min and ≥600min are in the final database, respectively.

### Smoking behavior assessment

Smoking behavior data is extracted from the SMQ dateset, which provides survey participants' cigarette use history, age of start, use in the last 30 days, cigarette brand, sub-brand, and other relevant details. For adults 18 years of age or older, trained interviewers ask questions at home using a computer-assisted Personal Interview (CAPI) system. The codes "1" and "2" represent whether or not you smoke at the current stage.

### Covariate

Covariates included gender, age, race, education, marital status, and income-poverty ratio. A total of 2,015 participants were divided into three age groups: 20-39 years, 40-59 years, and >60 years. Race is divided into Hispanic, non-Hispanic white, non-Hispanic black, non-Hispanic Asian, and other races. The education level is divided into below high school, high school and above high school. Marital status was divided into cohabitation, married living alone (widowed, divorced, separated) and never married. The poverty ratio is a measure of poverty measured by dividing household income by the survey year. In this study, the poverty ratio was used to create two income conditions, poor (<1.3) and middle income (≥1.3) [20].

### Statistical analysis

We used Microsoft Excel 2010 to extract and merge the raw data and exclude missing and useless (rejected, don't know) items. The database includes adults 20 years of age and older with complete information. For the purpose of this study, we tested the significance of the differences in covariates between the "smoking" and "non-smoking" groups. Rank sum test was used for quantitative variables and chi-square test for categorical variables. We used a binary logistic regression model to analyze the relationship between different types of physical activity and smoking behavior. All data were analyzed using the Statistical Product and Service Solutions (SPSS) version 26.0, and a *P*-value less than 0.05 was considered statistically significant (bilateral test). Variables that were statistically significant in the univariate analysis were included in the stepwise binary logistic regression analysis. In univariate analysis, all covariables (*P* < 0.05) except gender (*P*=0.077) were statistically significant. In the significance test of measurement data, age *P* < 0.05 (variance was not homogeneous), *P*(bilateral) < 0.001, the difference was not statistically significant. Therefore, age and sex were not excluded as confounding factors in logistic regression analysis. A-entry=0.05 and a-exit=0.10 were used to select and exclude confounding variables.

When analyzing the relationship between physical activity at work and smoking behavior. We took work physical activity as the independent variable (1=yes, 2=no) and smoking (1=smoking, 2=no smoking behavior) as the dependent variable. To exclude the effect of confounding variables, we built the following models: Model I: Only the independent variable work physical activity was adjusted. Model II: Adjusted for independent variables in model I plus demographic variables (race, education, marital status, income-poverty ratio). Model III: Adjusted for model II plus variables for recreational physical activity, commuting physical activity, and sedentary behavior.

When analyzing the relationship between recreational physical activity and smoking behavior. We took recreational physical activity as the independent variable (1=yes, 2=no) and smoking (1=smoking, 2=no smoking behavior) as the dependent variable. To exclude the effect of confounding variables, we built the following models: Model IV: Only the independent variable recreational physical activity was adjusted. Model V: Adjusted for independent variables in model IV plus demographic variables (race, education, marital status, income-poverty ratio). Model VI: Adjusted for model V plus variables for work physical activity, commuting physical activity, and sedentary behavior.

When analyzing the relationship between commuting physical activity and smoking behavior. We took commuting physical activity as the independent variable (1=yes, 2=no) and smoking (1=smoking, 2=no smoking behavior) as the dependent variable. To exclude the effect of confounding variables, we built the following models: Model VII: Only the independent variable commuting physical activity was adjusted. Model VIII: Adjusted for independent variables in model VII plus demographic variables (race, education, marital status, income-poverty ratio). Model IX: Adjusted for model VIII plus variables for work physical activity, recreational physical activity, and sedentary behavior.

On the relationship between sedentary behavior and smoking behavior. We took sedentary behavior as the independent variable (1=yes, 2=no) and smoking (1=smoking, 2=no smoking) as the dependent variable. To exclude the effect of confounding variables, we built the following model: Model X: Only the sedentary behavior of the independent variable was adjusted. Model XI: Adjusted for independent variables in model X plus demographic variables (race, education, marital status, income-poverty ratio). Model XII: Adjusted for model XI plus variables for work physical activity, recreational physical activity, and commuting physical activity.

## Results

### Demographic characteristics

The study included 2,015 adults aged 20 years or older in the 2017-2018 U.S. National Nutrition Examination Survey cycle who completed data on physical activity, smoking, and other demographic information. There were statistically significant differences in covariates and independent variables such as race, educational level, marital status and income poverty ratio between the smoking group and the non-smoking group (See Table 1).

**Table 1:** Demographic characteristics of adults aged 20 years and older, by smoking behavior

**Characteristics, n%**	**Sample Capacity**	**Smoking**	**Non-Smoking**	**Test statistics**	***P***
	*N*=2015	*n*=845	*n*=1170		
**Gender**				3.119^a^	0.077
Male	1233 (48.81)	498 (43.35)	735 (51.05)		
Female	782 (51.19)	347 (56.65)	435 (48.95)		
**Age Group**				208.422^b^	<0.001^***^
20-39	516 (30.53)	321 (21.14)	195 (34.39)		
40-59	594 (32.07)	297 (36.50)	297 (30.24)		
≥60	905 (37.40)	227 (42.36)	678 (35.37)		
**Race**				35.141^a^	<0.001^***^
Hispanic	361 (21.31)	119 (18.08)	242 (22.64)		
Non-Hispanic White	932 (37.41)	379 (46.41)	553 (33.71)		
Non-Hispanic Black	446 (22.61)	229 (21.22)	217 (23.18)		
Non-Hispanic Asian	141 (13.42)	50 (7.93)	91 (15.72)		
Other	135 (5.25)	68 (6.36)	67 (4.75)		
**Education**				11.436^a^	0.003^**^
Below high school	442 (17.46)	202 (16.27)	240 (17.96)		
High school	568 (24.25)	259 (24.11)	309 (24.30)		
Post high school	1005 (58.29)	384 (59.62)	621 (57.74)		
**Marital Statues**				61.361^a^	<0.001^***^
Cohabitation	1166 (59.95)	445 (55.49)	721 (61.78)		
Married living alone	556 (22.78)	216 (28.74)	340 (20.33)		
Not married	293 (17.27)	184 (15.77)	109 (17.89)		
**Income to Poverty**				85.973^a^	<0.001^***^
Impoverished	686 (27.59)	385 (28.74)	301 (27.12)		
Moderate income	1329 (72.41)	460 (71.26)	869 (72.88)		
**Work physical activity**				12.959^a^	<0.001^***^
Yes	1064 (48.09)	486 (50.54)	578 (47.08)		
No	951 (51.91)	359 (49.46)	592 (52.92)		
**Recreational physical activity**				6.576^a^	0.010^*^
Yes	808 (47.73)	311 (43.68)	497 (49.39)		
No	1207 (52.27)	534 (56.32)	673 (50.61)		
**Commuting physical activity**				13.631^a^	<0.001^***^
Yes	455 (23.09)	225 (21.14)	230 (23.90)		
No	1560 (76.91)	620 (78.86)	940 (76.10)		
**Sedentary Behavior**				6.829^a^	0.009^**^
<600min	1690 (84.92)	730 (81.50)	960 (86.32)		
≥600min	325 (15.08)	115 (18.50)	210 (13.68)		

^*^P < 0.05, ^**^P < 0.01, ^***^P < 0.001

^a^chi-square test

^b^Kruskal-Wallis-test

### Association between physical activity at work and smoking behavior

In logistic regression analysis, model I (without adjusting any confounding variables) showed an odds ratio (OR) of 1.396 (95%Cl:1.245-1.566) for the association between work physical activity and smoking behavior. Model II (adjusted for variables of gender, race, education, marital status, and income-poverty ratio) shows that OR=1.143 (95%Cl:1.009-1.296). Model III (adjusted for all confounding variables) shows OR=1.135 (95%Cl:0.999-1.289). The findings suggest that after adjusting for all confounding factors, physical activity at work is close to significant with smoking behavior, and physical activity at work may be a potential risk factor for smoking behavior. Weekly physical activity at work was associated with a 28.9 percent increased risk of smoking (*P* < 0.01) (See Table 2).

**Table 2:** Logistic regression analysis of physical activity at work and smoking behavior

***Mode***	***b***	**SE**	***Wald***	***P***	**OR(95%Cl)**
I^a^	0.334	0.058	32.587	<0.001^***^	1.396 (1.245-1.566)
II^b^	0.134	0.064	4.411	0.036^*^	1.143 (1.009-1.296)
III^c^	0.126	0.065	3.753	0.053	**1.135 (0.999-1.289)**

*P < 0.05, ***P < 0.001

^a^Only the independent variable work physical activity was adjusted

^b^Adjustments were made for independent variables in Model I plus demographic variables (gender, race, education, marital status, and income-poverty ratio)

^c^Adjustments were made for Model II with the addition of variables for recreational physical activity, commuting physical activity, and sedentary behavior

### Association between recreational physical activity and smoking behavior

In logistic regression analysis, Model IV (without adjusting any confounding variables) showed that the odds ratio (OR) of the association between recreational physical activity and smoking behavior was 0.828 (95%Cl:0.737-0.931). Model V (adjusted for variables of gender, race, education, marital status, and income-poverty ratio) shows that OR=0.729 (95%Cl:0.639-0.832). Model VI (adjusted for all confounding variables) shows OR=0.695 (95%Cl:0.608-0.795). The findings showed that recreational physical activity was a protective factor for smoking behavior after adjusting for all confounding factors. Weekly recreational physical activity was associated with a 39.2-20.5% lower risk of smoking behavior (*P* < 0.01) (See Table 3).

**Table 3:** Logistic regression analysis of recreational physical activity and smoking behavior

***Mode***	***b***	**SE**	***Wald***	***P***	**OR(95%Cl)**
IV^a^	-0.188	0.059	10.054	0.002^**^	0.828 (0.737-0.931)
V^b^	-0.315	0.067	22.016	<0.001^***^	0.729 (0.639-0.832)
VI^c^	-0.364	0.068	28.173	<0.001^***^	**0.695 (0.608-0.795)**

**P < 0.01, ***P < 0.001

^a^Only the independent variable recreational physical activity was adjusted

^b^Adjustments were made for independent variables in Model IV plus demographic variables (gender, race, education, marital status, and income-poverty ratio)

^c^Adjustments were made for Model V plus variables for physical activity at work, commuting physical activity and sedentary behavior

### Association between commuting physical activity

In logistic regression analysis, Model VII (without adjusting any confounding variables) showed an odds ratio (OR) of 1.550 (95%Cl:1.355-1.773) for the association between commuting physical activity and smoking behavior. Model VIII (adjusted for variables of gender, race, education, marital status, and income-poverty ratio) shows that OR=1.214 (95%Cl:1.048-1.405). Model IX (adjusted for all confounding variables) shows OR=1.278 (95%Cl:1.101-1.484). The findings showed that after adjusting for all confounding factors, commuting physical activity was a risk factor for smoking behavior. Weekly commuting physical activity was associated with an increased risk of smoking by 10.1-48.4% (*P* < 0.01) (See Table 4).

**Table 4:** Logistic regression analysis of commuting physical activity and smoking behavior

***Mode***	***b***	**SE**	***Wald***	***P***	**OR(95%Cl)**
VII^a^	0.438	0.069	40.733	<0.001^***^	1.550 (1.355-1.773)
VIII^b^	0.194	0.075	6.729	0.009^**^	1.214 (1.048-1.405)
IX^c^	0.246	0.076	10.422	0.001^**^	**1.278 (1.101-1.484)**

**P < 0.01, ***P < 0.001

^a^Only the independent variable commuting physical activity was adjusted

^b^Adjusted for independent variables in Model VII plus demographic variables (gender, race, education, marital status, and income-poverty ratio)

^c^Adjusted for model VIII with the addition of variables for work physical activity, recreational physical activity, and sedentary behavior

### Association between sedentary behavior and smoking behavior

In logistic regression analysis, model X (without adjusting any confounding variables) showed that the odds ratio (OR) associated sedentary behavior with smoking behavior was 1.479 (95%Cl:1.265-1.729). Model XI (adjusted for variables of gender, race, education, marital status, and income-poverty ratio) shows that OR=1.363 (95%Cl:1.154-1.611). Model XII (adjusted for all confounding variables) shows that OR=1.319 (95%Cl:1.113-1.564). The results showed that after adjusting for all confounding factors, sedentary behavior ≥600min was a risk factor for smoking behavior. Sedentary behavior ≥600 minutes per day was associated with an 11.3-56.4% increased risk of smoking behavior (*P* < 0.01) (See Table 5).

**Table 5:** Logistic regression analysis of sedentary behavior and smoking behavior

***Mode***	***b***	**SE**	***Wald***	***P***	**OR(95%Cl)**
X^a^	0.392	0.080	24.162	<0.001^***^	1.479 (1.265-1.729)
XI^b^	0.310	0.085	13.275	<0.001^***^	1.363 (1.154-1.611)
XII^c^	0.277	0.087	10.231	0.001^**^	**1.319 (1.113-1.564)**

**P < 0.01, ***P < 0.001

^a^Only the independent variable was adjusted for sedentary behavior

^b^Adjustments were made for independent variables in model X plus demographic variables (gender, race, education, marital status, and income-poverty ratio)

^c^Adjusted for model XI with the addition of variables for work physical activity, recreational physical activity and commuting physical activity

## Discussion

In this study, we found that different types of physical activity were independently associated with smoking behavior in adults over 20 years of age. Work physical activity, commuting physical activity, and sedentary behavior might increase the risk of smoking behavior, and recreational physical activity may potentially decrease the risk of smoking behavior. Therefore, we will discuss "Work physical activity and smoking behavior," "Recreational physical activity and smoking behavior," "Commuting physical activity and smoking behavior," and "Sedentary behavior and smoking behavior," respectively, in the following texts.

### Work physical activity and smoking behavior

Nadell [21] found that higher weekly physical activity at work was associated with higher smoking behavior. A study by Willy showed that the more hours a smoking nurse aide worked per week, the less likely they were to stop smoking [22]. Our findings suggest that physical activity at work is a potential risk factor for smoking behavior and may increase the risk of smoking behavior by about 13.5%. Sports motivation may explain this problem in one way. Traditionally, physical activity is motivated by socializing and improving physical health and appearance. However, physical activity at work is not motivated by avoiding negative health problems, combating smoking, or improving physical appearance; it may be done out of necessity or compulsion [23]. Active natural physical exercise is associated with positive mood and fewer daily stressors, leading to lower depression scores [24]. Engaging in inactive physical activity at work can make your mood and mood worse [21]. Higher negative emotions were associated with increased smoking behavior [25]. In addition, engaging in non-active work physical activity may increase the risk of sitting and obesity, and at the same time, may not have more energy for exercise [26], which may also be associated with increased smoking behaviors.

### Recreational physical activity and smoking behavior

In previous studies, Patel [27] has suggested that smokers tend to be less physically active or thinner. Holmen [28] has shown that the frequency of physical exercise is negatively correlated with smoking behavior, and smokers are more likely not to participate in exercise. Conway [29] has also reported that smoking is associated with lower exercise levels and lower physical endurance. This study supports the conclusion of previous studies that recreational physical activity is a protective factor for smoking behavior, reducing the risk of smoking behavior by about 30.5%. Studies have shown that moderate-intensity exercise is effective in reducing cravings for reward components, thereby delaying cravings for the anticipated reward or snack of smoking [30, 12]. Regular physical exercise before quitting smoking can reduce the occurrence of smoking behavior and put smokers in a favorable state to quit smoking [31]. People who regularly engaged in recreational physical activity may be more likely to experience the positive effects of exercise on their health [32], and they may place more emphasis on their health and thus resist smoking behaviors. Smoking can adversely affect pulmonary functions and endurance quality [33], which can reduce the performance of smokers and make them more likely to feel tired during exercise, leading to a decrease in physical activity among smokers. In addition, engaging in recreational physical activity can make the body release endorphins and dopamine, which can make people have a positive emotional experience, which may also be one of the reasons for reducing smoking behavior.

### Commuting physical activity and smoking behavior

Commuting physical activity and smoking may have little been mentioned in previous studies, with a 2010 study showing no significant correlation between commuting activity and smoking [34]. Our findings may be somewhat different, suggesting that commuting to physical activity increases the risk of smoking behavior. Studies have shown that a majority of people in Italy support the introduction of a smoking ban in cars, especially in cars carrying children [35]. In some parts of the United States, smoke-free vehicle laws have also been enacted to protect the physical health of young people [36]. With the implementation of these laws, since smoking is prohibited in cars, smokers may smoke while walking or riding bicycles, or choose to walk and ride bicycles for the sake of smoking. At the same time, when walking and riding a bicycle, there is no legal constraint, which will create an environment that is convenient for smoking, which will make people more prone to smoking. Therefore, if you want to quit smoking or control smoking behavior, you should try to avoid creating an environment where you can smoke.

### Sedentary behavior and smoking behavior

In the analysis of sedentary behavior and smoking behavior, our results differ from the study by Vanessa, which found no difference in sedentary behavior between smokers and non-smokers. Our findings support studies by Efendi [10] and Lee [11], which show that smokers tend to be less physically active and engage in more sedentary behavior. At the same time, sedentary behavior is significantly associated with mental illness, and prolonged sitting increases the risk of anxiety, depression, and suicide [37]. While another study has shown that for smokers, smoking can bring many perceived benefits, including enhanced mood, reduced anxiety, and weight control [38]. The negative effects of sedentary behavior may induce some people to smoke for relief. People who are regularly physically active are more confident in their ability to control their smoking, while people who have taken action to change their smoking behavior are more confident in their ability to exercise [39]. This may enable people with relatively short periods of sedentary behavior to control their smoking behavior more. Although sedentary behavior is less harmful than smoking [40], sedentary behavior can lead to smoking behavior, and smoking has a significant additive effect with low levels of physical activity [41], so it is necessary to avoid prolonged sedentary time.

### Limitations and perspectives of this study

This study is innovative and significant to some extent, but has the following limitations: 1) We are not perfect in excluding confounding factors: The causes of smoking behavior are multi-factorial, including a variety of genetic, biological, environmental and social factors, and this study could not exclude all influencing factors.

Future research should, in our opinion, address the aforementioned issues. To account for additional confounding variables, it is advisable to include as many covariates as possible. Furthermore, even though we have classified physical activity, it is a complex behavioral activity that is influenced by many variables. Therefore, if the evaluation of physical activity could be more precise, we believe that this study could have significant implications for future research.

## Conclusions

After adjusting for all confounding factors, physical activity at work was a potential risk factor for smoking behavior. Recreational physical activity was a protective factor for smoking behavior. Commuting physical activity and sedentary behavior are risk factors for smoking behavior. There is a strong association between physical activity and smoking behavior, but different types of physical activity have different associations with smoking behavior. Therefore, when physical activity is used for tobacco control, it cannot be confused, and it is necessary to pay attention to the type and environment of physical activity. Recreational physical activities should be appropriately increased, sedentary behavior should be reduced, and smoking prohibit environment should be expanded as far as possible to achieve better clinical intervention effects.

## Data Availability

The datasets generated and/or analyzed during the current study are available in the [NHANES] repository, [NHANES Questionnaires, Datasets, and Related Documentation (cdc.gov)]. Raw data supporting the obtained results are available at the corresponding author.

## Abbreviations

NHANES: National Health and Nutritional Examination Survey
SPSS: Statistical Product and Service Solutions
WHO: World Health Organization
GPAQ: Global Physical Activity Questionnaire
SMQ: Smoking-Cigarette Use Questionnaire
OR: Odds Ratio
CI: Confidence Interval
P: *P*-Value
CAPI: Computer-Assisted Personal Interviewing
